# Establishment of an efficient regeneration system of ‘ZiKui’ tea with hypocotyl as explants

**DOI:** 10.1038/s41598-024-62319-1

**Published:** 2024-05-21

**Authors:** Jiongyi Jin, Yulu Chen, Ju Cai, Litang Lv, Xiaofang Zeng, Jianrong Li, Sumeera Asghar, Yan Li

**Affiliations:** 1 The Key Laboratory of Plant Resources Conservation and Germplasm Innovation in Mountainous Region (Ministry of Education), Institute of Agro-Bioengineering and College of Life Sciences, Guizhou University, Guiyang, 550025 China; 2 College of Tea Sciences, Guizhou University, Guiyang, 550025 China

**Keywords:** Plant breeding, Plant hormones, Plant reproduction

## Abstract

Zikui (*Camellia sinensis* cv. *Zikui*) is a recently discovered cultivar of local purple tea in Guizhou, China. It is a purple leaf bud mutation material of Meitan Taicha (*Camellia sinensis* cv. ‘Meitan-taicha’) ‘N61’ strain, which is an important local germplasm resource in Guizhou. It is also a model plant for the study of anthocyanins, but the limited germplasm resources and the limitation of traditional reproduction hinder its application. Here, an efficient regeneration system is established by using hypocotyl as explants for the first time. Different plant growth regulators (PGRs) are evaluated during different regeneration processes including callus and root induction. According to our findings, using the optimal disinfection conditions, the seed embryo contamination rate is 17.58%. Additionally, the mortality rate is 9.69%, while the survival rate is measured as 72.73%. Moreover, the highest germination rate of 93.64% is observed under MS + 2.40 mg/L GA_3_ medium conditions. The optimal callus induction rate is 95.19%, while the optimal adventitious bud differentiation rate is 20.74%, Medium with 1.6 mg/L IBA achieved 68.6% rooting of the adventitious shoots. The survival rate is more than 65% after 6 days growth in the cultivated matrix. In summary, our research aims to establish a regeneration system for Zikui tea plants and design a transformation system for tea plant tissue seedlings. This will enable transfer of the target gene and ultimately facilitate the cultivation of new tea varieties with unique characteristics.

## Introduction

Tea plants (*Camellia sinensis* L.) are extensively cultivated worldwide, and tea is also among the top three non-alcoholic beverages globally^1^. Purple leaf tea is a type of tea germplasm resource characterized by purple buds and leaves, and it contains a high amount of anthocyanin. Anthocyanins exhibit significant antioxidant properties, thereby enhancing the health benefits of purple bud varieties in tea production. Consequently, it is a crucial area of concentration in the breeding and investigation of tea tree^2^. It is a dire need to establish the protocols for successful tissue culture propagation of the Zikui tea plant.

Zikui is a new line of purple tea tree with unique local characteristics from the Meitan Taicha population, which is known for being an excellent germplasm resource of tea trees in Guizhou. Zikui tea is a clonal shrubby small-leaved variety. The mature leaves are dark green with a long growth period, soft leaf quality, flat leaf surface, and flat or slightly wavy leaf edge. The purple buds have a reddish-purple color, medium hairs, and a stout, short appearance. Young buds, leaves color is also purple red, buds and leaves cold resistance, drought resistance is strong, wide adaptability. The leaves of spring tea contain 5.40% free amino acids, 17.30% tea polyphenols, 3.10% caphine and 12.85% catechins. The green tea made from purple tea tree has a mellow taste, a long-lasting aroma and a little bitter^3^. Anthocyanins are pigments soluble in water^4^, possess numerous beneficial properties including free radical scavenging^5^, anticancer effects^6^, and prevention of cardiovascular and cerebrovascular disorders^7^. Its health function has also become one of the important qualities of purple tea breeding^8^. The anthocyanins in purple tea are rich in resources, safe and non-toxic, with diverse biological activities, and have become a hot research and development topic in the field of food and health products^9^. Zikui trees have an exceptionally elevated concentration of anthocyanins, reaching 4.97 mg/g. This amount surpasses that of other tea tree varieties by more than threefold^10^. Additionally, the antioxidative capability of this compound is remarkable, being 50 times stronger than vitamin E and 20 times more potent than vitamin C. Anthocyanins can be extracted from the Zikui tea tree as well^11–13^. Moreover, the anthocyanin content of purple tea tree reached 0.5–1.0% of dry matter weight^14^. In addition, anthocyanins have attracted increasing attention from researchers due to their various bioactive effects such as antibacterial, salt resistance, and lipid reduction, as well as their beneficial effects on cardiovascular diseases, eye diseases, neurodegenerative diseases, and cancer^15,16^. To address the issue of limited tea seedlings, one possible solution is to integrate the Zikui tissue regeneration system with various biotechnologies, including genetic engineering^17^. This combination can offer sterile resources and serve as a valuable technical resource for future investigations into the mechanisms underlying the transformation of tea into a purple variant. The important prerequisite for establishing a genetic transformation system for tea trees is to establish a regeneration system for tea tree differentiation through tissue culture technology^18^. The genetic transformation of Zikui tea trees can be greatly influenced by the presence of the exceptional and uncommon compound known as anthocyanins. This particular substance holds remarkable scientific research value due to its rarity and unique characteristics.

For the purpose of investigating the propagation mechanism in tea trees, a novel regeneration system is successfully developed using hypocotyl as explants. Through tissue culture, we achieved good treatment conditions and develop suitable culture medium formulations, thereby enhancing the research achievements of tea plant in vitro culture technology. As a next step, the combination of tea plant tissue culture and transgenic technology will be explored to establish an advanced tea plant tissue culture seedling transformation system. This system would facilitate the transfer of target genes using the developed regeneration system.

## Materials and methods

### Plant materials

Efficient regeneration system is developed by inducing selected Zikui seed embryos, collected from October to November, as explants, with various plant growth regulators (PGR). To facilitate the growth of fully adapted plants within their respective environments, this study focused on exploring the mechanisms involved in seed embryo germination, hypocotyl callus formation, differentiation, the adventitious buds are grown vigorously, rooting processes, and transplanting seedlings.

### Seed disinfection

After harvesting, the Zikui seeds are extracted by removing the outer peel and then removing the seed shells using a sheller. Subsequently, high-quality seeds free from pollution are chosen and soaked in a beaker containing a solution of dishwashing liquid for a duration of 8 min. The seeds are stirred gently and rinsed with tap water for over 10 min. Subsequently, they are soaked in a carbendazim solution with a concentration of 0.50% for 10 min, with continuous stirring. Following the soaking, the seeds are rinsed again with tap water for more than 10 min, while being continuously stirred. Finally, the seeds are placed on a super-clean table for further use. The seeds are placed on the clean bench for subsequent use. The experiment included two disinfectants: 75% ethanol disinfection for 1, 2, and 3 min, and 20% sodium hypochlorite for 7, 10, and 13 min. This resulted in a total of 9 treatment groups. Following disinfection with 75% ethanol, the seeds are rinsed three times with sterile water and further disinfected using 20% sodium hypochlorite. To mitigate any potential harm caused by residual disinfection agents, the seeds undergo a thorough rinsing process, consisting of more than seven cycles of sterile water. Following the oscillation, the water from the seed is removed using a sterile blotting paper. The seed embryo is then extracted using a sterile blade and subsequently placed into the seed embryo germination medium. The contamination rate, survival rate, and mortality rate are calculated using Eqs. (1)–(3).

1 $${\text{Contamination rate }}\left( \% \right) \, = \, \left( {{\text{Number of contaminated explants/}}{\text{total number of inoculated explants}}} \right) \, \times {1}00\% ,$$

2 $${\text{Survival rate }}\left( \% \right) \, = \, \left( {{\text{Number of active explants}}/{\text{total number of explants inoculated}}} \right) \times {1}00\% ,$$

3 $${\text{Mortality rate }}\left( \% \right) \, = \, \left( {{\text{Number of non{-}viable explants}}/{\text{total number of explants inoculated}}} \right) \times {1}00\% .$$

### Determine the suitable medium for seed embryo germination

The above sterilized embryos are inoculated into seed embryo germination medium with different GA_3_ concentrations, which was MS as the base medium, and the seed embryo germination medium with different GA_3_ concentrations is added into the MS as the base medium, and 4 treatment groups with different GA_3_ ratio (0.00–3.60 mg/L) were added. Each treatment group is repeated 3 times and 110 doses per dose. The germination of seeds is observed and recorded, and the germination number is recorded after 60 days as Eq. (4).

4 $${\text{Seed embryo germination rate }}\left( \% \right) \, = \, \left( {{\text{number of seed embryo germination}}/{\text{total number of seed embryo inoculation}}} \right) \, \times {1}00\% .$$

### Dark culture days of hypocotyl differentiation are determined

After the hypocotyl of the seedlings in the above germination medium grew out, the hypocotyl of the seedlings cut into small segments of about 1 cm, which are used as explants for callus induction and differentiation, and inoculated into WPM + 2.00 mg/L 6-BA + 0.10 mg/L NAA callus and differentiation medium. After being cultured for different periods (0, 7, 14 days) in a dark environment, they are transferred to a light condition for further culture. The experiment consisted of three treatment groups, with each group being repeated three times, resulting in a total of 90 inoculations per group. The callus induction rate, and adventitious bud differentiation rate are measured by Eqs. (5) and (6).

5 $${\text{Callus induction rate after }}0\;{\text{day}}/{7}\;{\text{days}}/{14}\;{\text{days dark culture }}\left( \% \right) \, = \, \left( {{\text{Number of callus induced by explants after }}0\;{\text{day}}/{7}\;{\text{days}}/{14}\;{\text{days dark culture}}/{\text{total number of explants inoculated}}} \right) \, \times {1}00\% ,$$

6 $${\text{Adventist bud differentiation rate after }}0\;{\text{day}}/{7}\;{\text{day}}/{14}\;{\text{days dark culture }}\left( \% \right) \, = {\text{Number of callus differentiation buds after }}0\;{\text{day}}/{7}\;{\text{days}}/{14}\;{\text{days dark culture}}/{\text{total number of explants inoculated}}) \, \times {1}00\% .$$

### Callus and adventitious bud differentiation were induced

The hypocotyl of the seedlings are cut into small segments measuring approximately 1 cm. These segments are then used as explants for callus induction and differentiation. They are inoculated into the hypocotyl differentiation medium, which contained varying proportions of plant growth regulators. The base medium for axial differentiation is WPM. Six treatment groups are formed by using various concentrations of IBA (0.10, 0.30 mg/L), NAA (0.10, 0.30 mg/L), and 2, 4-D (0, 10, 0.30 mg/L) in a 2.00 mg/L 6-BA solution. Each treatment group is repeated three times, resulting in a total of 90 doses. After a period of 30 days, the growth state and number of callus are observed. Subsequently, at the 60 day mark, the growth of adventitious buds and the rate of explant differentiation are observed once more.

5 $${\text{Callus induction rate }}\left( \% \right) \, = \, \left( {{\text{number of callus induced by explants}}/{\text{total number of explants inoculated}}} \right) \, \times {1}00\% ,$$

6 $${\text{Adventitious bud differentiation rate }}\left( \% \right) \, = \, \left( {{\text{number of callus differentiation buds}}/{\text{total number of explants inoculated}}} \right) \, \times {1}00\% .$$

### Effect of dark culture on hypocotyl Browning

The hypocotyl is inoculated with WPM + 2.00 mg/L 6-BA + 0.10 mg/L NAA callus and differentiation medium, then cultured in dark environment for different periods (0, 7, 14 days), and then transferred to light for further culture. The experiment is divided into 3 treatment groups, each group is repeated 3 times, and each group is inoculated 90 times. The browning rate is measured as Eq. (7).

7 $${\text{Browning rate }} = \, \left( {{\text{Browning number of explants}}/{\text{total number of inoculated explants}}} \right) \, \times {1}00\%.$$

### Strengthen seedlings and take root

The adventitious buds are cut from the callus. The seedlings are inoculated and cultured in MS + 2.00 mg/L 6-BA + 0.60 mg/L IBA medium. The lower end of a robust, rootless seedling is measured approximately 5 cm in height and is diagonally cut from the strong seedling medium. It is then soaked in a sterile solution of 60.00 mg/LIBA for 8 min and inoculated into the rooting medium. The aim is to determine the optimal concentration of 1/2 MS + 1.60 mg/L IBA for root development.

### Transplanting seedlings

The sterile seedling are removed from the tissue culture to the greenhouse, and the bottle cap is opened during the process of hardening. The plant hardening time is 6 days under natural light. After it, the plants with the medium are rinsed and transplanted into a prepared V yellow loam: V vermiculite = 2:1 transplanting medium.

### Plant ethics statement

Zikui samples are collected from the Agricultural Bioengineering Research Institute of Huaxi District, Guiyang City, Guizhou Province (latitude 26° 11′–26° 34′ N, longitude 106° 27′–106° 52′ E), and the Tea Research Institute of Meitan County (latitude 27° 20′ N, longitude 107° 15′ E) Guizhou Province, China, between October and November 2019. The samples are collected by Professor Li Yan and Researcher Zhou Guolan. It is in the custody of Professor Li Yan of Guizhou University. The research is carried out at the Institute of Agricultural Bioengineering. The study complies with relevant institutional, national and international guidelines and legislation.

### Statistical analysis

The sampling and inoculation of explants are randomly conducted. The experimental data are collected and summarized using Microsoft Excel. After converting the original data, ANOVA and LSD multiple comparison analysis are performed on the experimental data using SPSS22.0.

### Culture conditions

In the process of regenerating Zikui tea tree culture, WPM is used as the base medium for cotyledon callus differentiation, while MS is used as the base medium for the remaining steps. For callus differentiation, the composition of the medium included 29.78 g/L of WPM powder, 10.00 g/L of sucrose, and 1.00 g/L of AGAR. During rooting induction, the medium consisted of 2.22 g/L of MS powder, 2.50 g/L of plant gel, and 15.00 g/L of sucrose. For other processes, the medium contained 4.43 g/L of MS powder, 30.00 g/L of sucrose, and 8.00 g/L of AGAR. The pH value of the medium is maintained between 5.80 and 6.00. The tissue culture room is set to a light intensity of 2000 lx, a temperature of 23 ± 2 °C, and a light time of 12 h/day.

## Result

### Effect of disinfection time on survival rate of seed embryo

The establishment of a germ aseptic system involves obtaining sterile hypocotyl and providing materials for the construction of a subsequent regeneration system. Research has demonstrated that varying the duration of disinfection significantly affects the rate of bacterial infection and mortality in seeds (Table 1). Through the comparison of 9 groups, it is observed that after 20 days, the pollution rate and mortality rate of 8 are lower compared to the other groups. The survival rate of this group reached 72.73%, which is the highest among all the groups and thus considered the most effective groups. Therefore, the disinfection time of Zikui tea seed embryos is 75% alcohol disinfection for 3 min and 20% sodium hypochlorite immersion for 10 min.

**Table 1:** Effect of disinfection time on survival rate of seed embryo.

75% Alcohol (min)	20% Sodium hypochlorite (min)	Number of explant (per unit)	Survival rate (%)	Pollution rate (%)	Mortality rate (%)
1	10	100	53.94 ± 7.09c	41.82 ± 2.41a	4.24 ± 1.39a
1	15	100	60.91 ± 1.57bc	31.82 ± 1.57bc	7.27 ± 0.00a
1	20	100	64.85 ± 2.10bc	30.61 ± 1.89bc	4.54 ± 1.57d
2	10	100	55.45 ± 2.73c	40.00 ± 2.41a	4.55 ± 0.91d
2	15	100	64.24 ± 5.01bc	28.18 ± 2.41c	7.58 ± 2.78bc
2	20	100	65.76 ± 2.29b	25.15 ± 2.78 cd	9.09 ± 0.91bc
3	10	100	67.88 ± 0.52ab	23.33 ± 1.05d	8.79 ± 1.39bc
3	15	100	72.73 ± 1.82a	17.58 ± 1.39e	9.69 ± 0.52b
3	20	100	66.67 ± 2.78b	15.45 ± 1.82e	17.88 ± 1.05a

The different letters in the table are significant at the 0.05 level.

Mean ± S.D.

When the disinfection time for 75% alcohol remained constant, the pollution rate gradually decreased with a 20% sodium hypochlorite increase in disinfection time. The lowest pollution rate recorded as 15.45%, while the highest pollution rate reached 41.82%. The survival rate initially increased and then decreased, with the lowest survival rate observed at 53.94% and the highest survival rate at 72.73%. When the disinfection time of 20% sodium hypochlorite remained unchanged, the contamination rate gradually decreased and the survival rate gradually increased with an increase in the disinfection time of 75% alcohol. Moreover, as the soaking time of the two disinfection reagents increased, the mortality rate also increased correspondingly, reaching a peak of 17.88%. Disinfection reagents have the capability to not only eliminate fungi and bacteria, but also have the potential to harm the structures, including the cells, of the material itself. The longer the disinfection time, the more damaged the seed embryo will be, and even die.

### The effect of GA_3_ on germination of seed embryos

**Figure 1:**
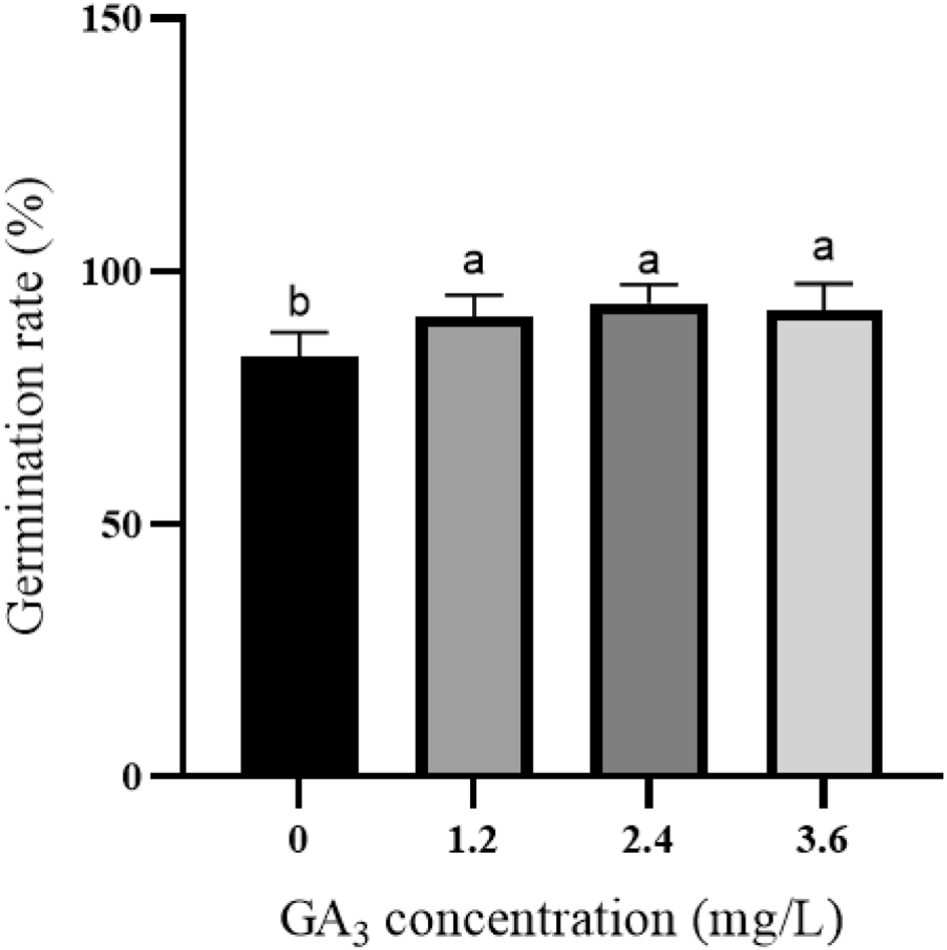
Effects of different GA_3_ concentrations on seed embryo germination. Different letters on the bars indicate significant differences with each other (P < 0.05), while same letters indicate the lack of significance, as determined by one-way analysis of variance (ANOVA) with Duncan’s post-test.

**Figure 2:**
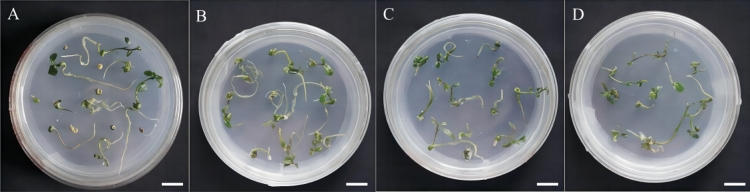
Growth of seed embryos germinated at different concentrations of GA_3_ for 60 days. (**A**) The growth of seed embryo inoculated on blank medium for 60 days; (**B**) The growth of seed embryo inoculated on MS + 1.20 mg/L GA_3_ medium for 60 days; (**C**) The seed embryo was inoculated on MS + 2.40 mg/L GA_3_ medium for 60 days; (**D**) Seed embryo was inoculated to MS + 3.60 mg/L GA_3_ medium for 60 days of growth. The ruler in the figure is 1 cm.

**Figure 3:**
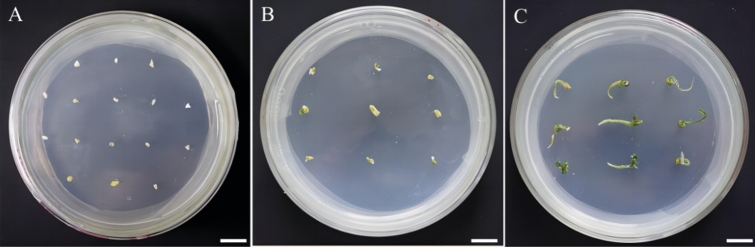
Growth of seed embryo germination induction at different time. (**A**) Growth after 10 days after culture; (**B**) Growth after 25 days of cultivation; (**C**) Growth after 45 days of cultivation; The ruler in the figure is 1 cm.

Gibberellin, a widely used and effective plant growth regulator, stimulates cell elongation and promotes plant growth and development. It is found that adding GA_3_ in the medium could effectively promote embryo germination and hypocotyl growth. (Fig. 1). In this study, the hypocotyl is chosen as the required explants. Group 3 exhibited the highest germination rate, likely due to the long hypocotyl and shorter root hair. The optimal treatment group in this experiment is group 3, which utilized MS + 2.4 mg/L GA_3_ as the seed embryo germination medium for Zikui Tea tree. GA_3_ is used to induce the breaking of seed dormancy and promote germination. It is observed that the germination rate is lower in MS medium without plant growth regulators after removing the cotyledon from the tea embryo. (Fig. 2A). The germination medium is supplemented with GA_3_ of varying concentrations and ratios (Table 2). The results showed a significant decrease in the germination period of seeds following the addition of GA_3_ (Fig. 3). The germination rate increased from 82.73 to over 90.91% when 1.20 mg/L GA_3_ was added (Fig. 2B). The germination rate for 2.4 mg/L GA_3_ was 93.64% (Fig. 2C), and for 3.6 mg/L GA_3_ it was 92.12% (Fig. 2D). There is no significant difference in the germination rate among the three treatment groups. Additionally, the germination period is shortened. However, the length of hypocotyl initially became shorter and then longer with increasing concentration. This suggests that the concentration of GA_3_ can have a certain effect on the elongation of the hypocotyl, with both high and low concentrations potentially impacting it.

**Table 2:** The effect of GA_3_ on germination of seed embryos.

GA_3_ (mg/L)	Inoculated number	Germination rate (%)	Germination at 60 days
–	110	82.73 ± 1.82b	Germination time is long, hypocotyl is short, root hair is long
1.20	110	90.91 ± 1.57a	Germination time is short, hypocotyl is short, root hair is long
2.40	110	93.64 ± 1.82a	Germination time is short, hypocotyl is long, root hair is short
3.60	110	92.12 ± 1.89a	Germination time is short, hypocotyl is short, root hair is short

The different letters in the table are significant at the 0.05 level.

Mean ± S.D.

### Effect of dark culture days on hypocotyl callus formation and differentiation

During callus induction, it is observed that dark culture conditions are more conducive to the dedifferentiation process into callus. On the other hand, under the influence of light, the formation of vascular bundles and other tissues are more prominent, leading to a disorder in the dedifferentiation process, which is found to be less favorable for callus generation. The growth rate of callus in plant tissue culture is faster in the absence of light. Therefore, this study determined the optimal dark culture days for hypocotyl differentiation in a medium with the same composition. And examined the relationship between the duration of dark culture days and the rate of differentiation (Table 3). The findings suggested a positive correlation between these two variables. However, it should be noted that the observed change range was relatively small. This could possibly be attributed to the fact that the medium used in this study might not the most optimal for hypocotyl differentiation. During the dark embryo development period, which ranged from 0 to 7 days and eventually reaches 14 days, there was a noticeable increase in the number of hypocotyl-induced calluses. In fact, the callus induction rate reached an impressive 93.64%. Hence, it can be concluded that the ideal duration for promoting hypocotyl differentiation through dark culture is 14 days.

**Table 3:** Effect of dark culture days on hypocotyl callus formation and differentiation.

Dark cultivation time (dayss)	Number of vaccinations	Callus induction (%)	Differentiation rate (%)
0	90	82.22 ± 2.22b	3.70 ± 0.64b
7	90	84.44 ± 2.22b	4.81 ± 0.64ab
14	90	93.64 ± 1.82a	5.93 ± 0.64a

The different letters in the table are significant at the 0.05 level.

Mean ± S.D.

### Effect of plant growth regulators on hypocotyl callus induction and differentiation

**Figure 4:**
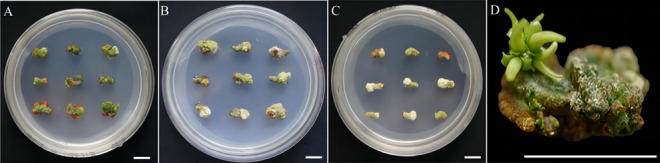
Differentiation of the hypocotyl at 60 days under different differentiation medium conditions. (**A**) The cotyl was inoculated on WPM + 2.00 mg/L 6-BA + 0.10 mg/L NAA medium for 60 days. (**B**) The hypocotyl was inoculated on WPM + 2.00 mg/L 6-BA + 0.10 mg/L IBA medium for 60 days; (**C**) Hypocotyl inoculation to WPM + 2.00 mg/L 6-BA + 0.10 mg/L 2, 4-D culture for 60 days. (**D**) Zoomed-in image of cotyl inoculation on WPM + 2.00 mg/L 6-BA + 0.10 mg/L NAA substrate for 60 days. The ruler in the figure is 1 cm.

**Figure 5:**
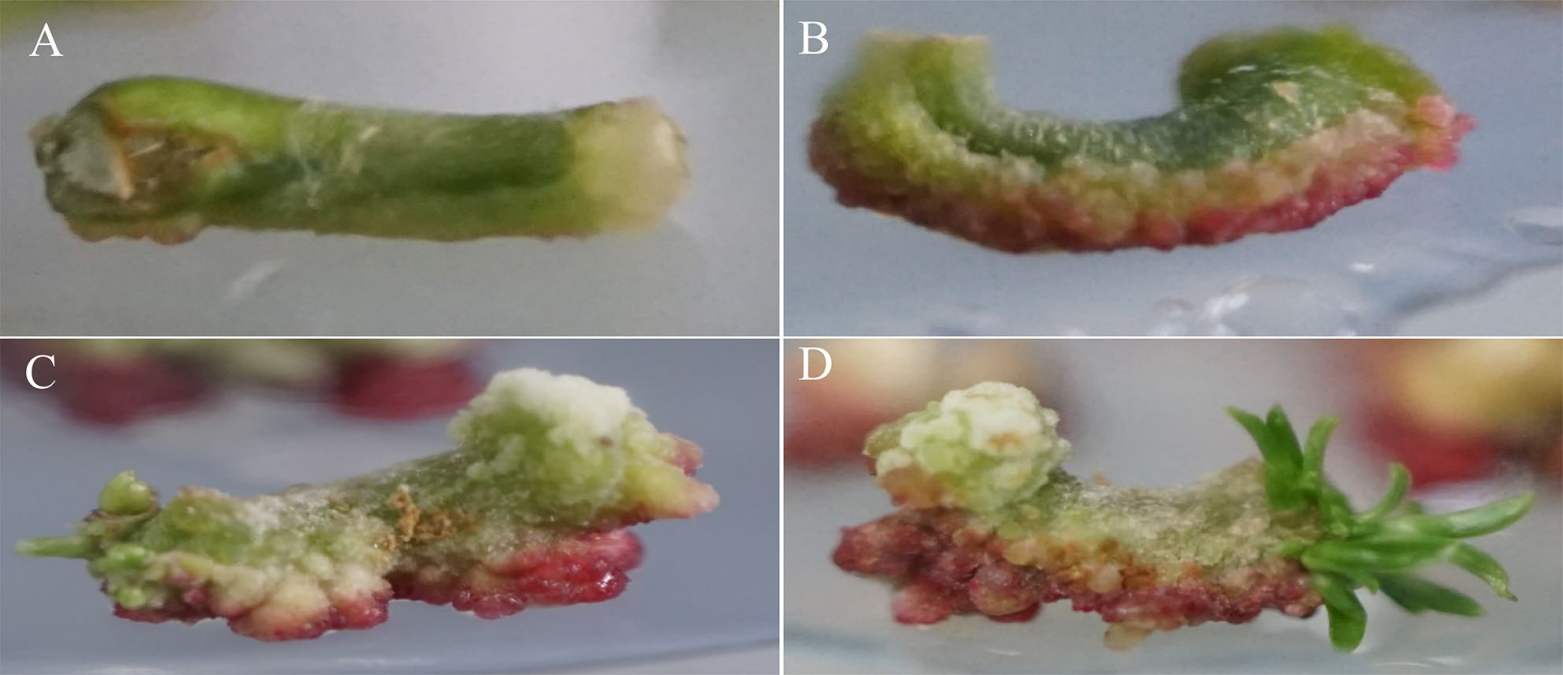
Hypocotyl callus formation and differentiation process in WPM + 2.00 mg/L 6-BA + 0.10 mg/L NAA. (**A**) Callus induction in hypocotyl culture at WPM + 2.00 mg/L 6-BA + 0.10 mg/L NAA for 0 days. (**B**) Callus induction in hypocotyl cultured with WPM + 2.0 mg/L 6-BA + 0.10 mg/L NAA for 20 days. (**C**) Callus induction in hypocotyl cultured at WPM + 2.00 mg/L 6-BA + 0.10 mg/L NAA for 40 days. (**D**) Callus induced by hypocotyl culture at WPM + 2.00 mg/L 6-BA + 0.10 mg/L NAA for 60 days. The ruler in the figure is 1 cm.

The induction of callus and buds are performed using the same medium. Different types and concentrations of plant hormones have different effects on callus induction and organ differentiation. The study showed that in six treatment groups (Table 4). The callus induction rate did not show a significant difference between group 1 and group 4. However, the differentiation rate of Group 1 is 20.74% (Fig. 4D), which is the highest among all the treatment groups. Based on factors such as callus growth status, it can be concluded that Group 1 performed the best among the 6 treatment groups (Fig. 5). Therefore, the callus induction and differentiation culture base of Zikui tea tree is WPM + 2.00 mg/L 6-BA + 0.10 mg/L NAA.

**Table 4:** Effect of plant growth regulators on hypocotyl callus induction and differentiation.

Processing condition	Inoculated number	Callus induction rate (%)	Callus number	Induction rate of bud differentiation (%)	Number of differentiated buds	Callus growth status
WPM + 2.00 mg/L 6-BA + 0.10 mg/L NAA	90	95.19 ± 0.64a	86	20.74 ± 0.64a	19	Green, compact structure, many adventitious buds
WPM + 2.00 mg/L 6-BA + 0.30 mg/L NAA	90	96.30 ± 1.70a	87	15.93 ± 1.70c	14	Green, compact structure, few adventitious buds
WPM + 2.00 mg/L 6-BA + 0.10 mg/L IBA	90	91.85 ± 1.28b	83	19.03 ± 1.11ab	17	Yellow-green, covering the surface of the cotyl, compact structure, few adventitious buds
WPM + 2.00 mg/L 6-BA + 0.30 mg/L IBA	90	97.04 ± 1.70a	88	18.15 ± 1.70b	16	Yellow-green, covering the surface of the cotyl, compact structure, few adventitious buds
WPM + 2.00 mg/L 6-BA + 0.10 mg/L 2, 4-D	90	90.00 ± 1.11bc	81	15.56 ± 1.11c	14	White and light green, covering the cotyl indicates a loose structure with few adventitious buds
WPM + 2.00 mg/L 6-BA + 0.30 mg/L 2, 4-D	90	88.89 ± 2.22c	80	8.52 ± 0.64d	6	White and light green, covering the cotyl indicates a loose structure with few adventitious buds

**Figure 6:**
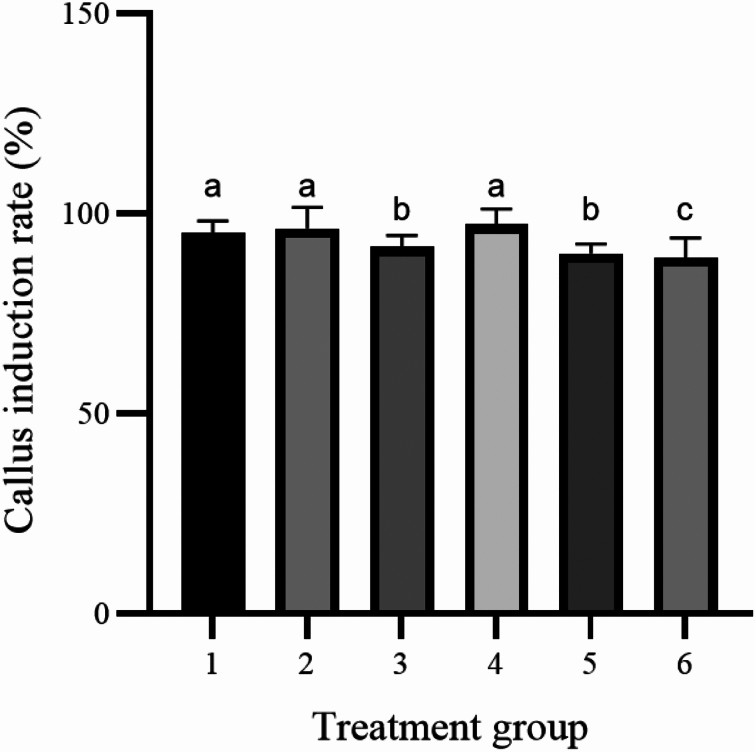
Effects of different proportions of plant growth regulators on callus induction. Different letters on the bars indicate significant differences with each other (P < 0.05), while same letters indicate the lack of significance, as determined by one-way analysis of variance (ANOVA) with Duncan’s post-test.

**Figure 7:**
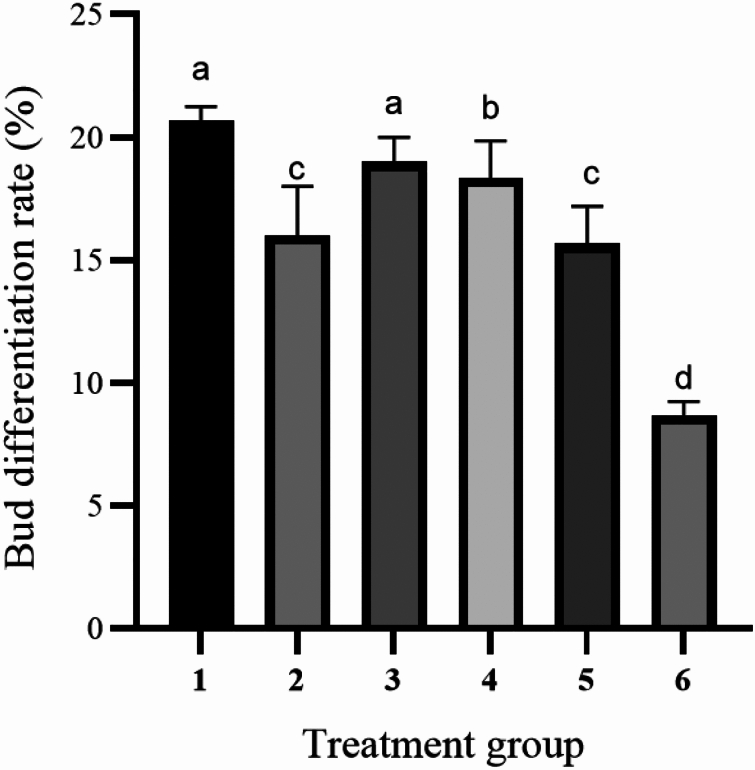
Effects of different proportions of plant growth regulators on adventitious bud differentiation. Different letters on the bars indicate significant differences with each other (P < 0.05), while same letters indicate the lack of significance, as determined by one-way analysis of variance (ANOVA) with Duncan’s post-test.

The results showed that the recovery rate of 6 groups ranged from 88.89% to 97.04%, and the callus induction rate of 6 groups is very high (Fig. 6). Under the conditions of 6-BA and NAA, the callus appeared green and the callus structured as tight, and the adviced-bud abundantly clustered with subsequent differentiation (Fig. 4A). The callus appeared yellowish green and have a tight structure, with a slight amount of browning observed at a later stage (Fig. 4B). Under the conditions of 6–BA and 2, 4-D, the callus displayed a mixture of green and white colors. The structure of the callus is loose, and there are only a few indefinite bud clusters present (Fig. 4C). As the concentrations of IBA, NAA and 2, 4-D regulators increased from 0.1 to 0.3 mg/L, the differentiation rates are found to be decreasing, and the concentration of 0.1 mg/L is more suitable for differentiation (Fig. 7).

### Effect of growth culture on the growth of adventitious buds

**Figure 8:**
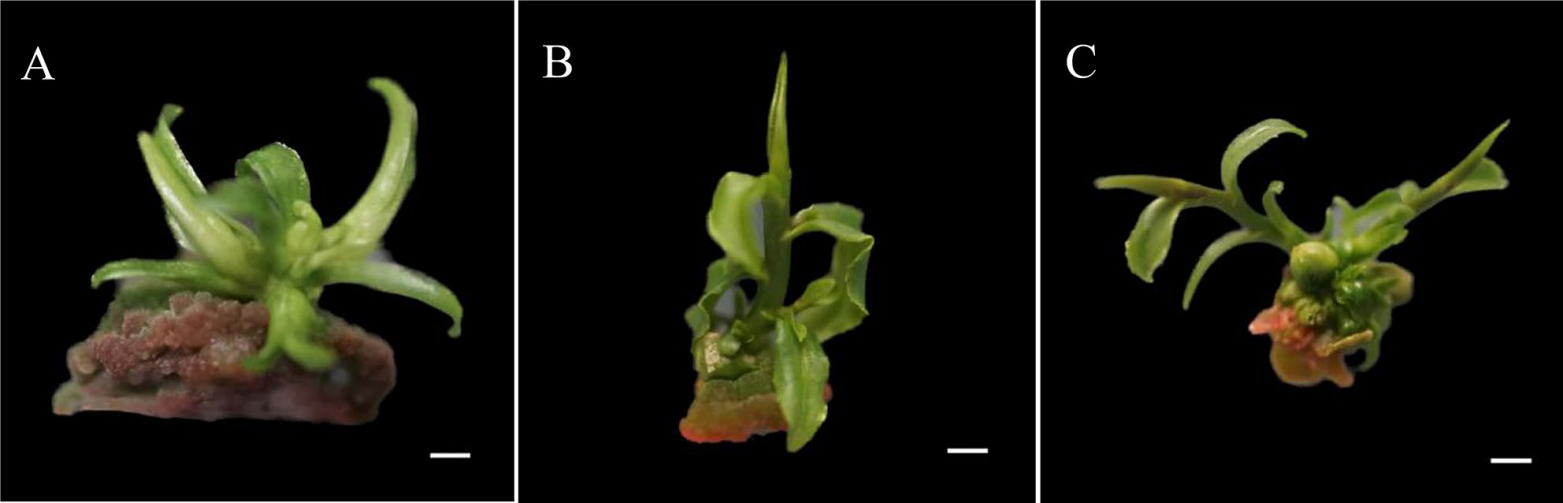
Growth of hypocotyl callus and adventitious bud culture. (**A**) Callus and adventitious buds induced by hypocotyl explants of Zikui tea; (**B**) The callus and indefinite buds of Zikui tea were grown and cultured for 15 days; (**C**) The callus and adventitious buds of Zikui tea were grown and cultured for 30 days. The ruler in the figure is 1 cm.

The hypocotyl explants of Zikui tea are induced by callus and adventitious bud differentiation, and the adventitious bud is very weak and only have leaves and almost no stem segment. If it is directly inoculated into the strong seedling medium for strong seedling culture, the regenerated adventitious buds are easy to die. If they are left alone and continued to be cultured on the original medium, the adventitious buds did not change much and grew slowly, probably because the nutrients and growth regulators in the medium have been consumed. Therefore, the callus with adventitious buds is selected from the medium with depleted nutrients and growth regulators (Fig. 8A). It is then inoculated into the same newly formulated medium for growth culture. After 15 days of culture, the adventitious buds significantly grew (Fig. 8B). When cultured to 30 days, the adventitious bud length was about 1 cm, and the growth was good and robust (Fig. 8C). After the growth culture of callus and adventitious buds, adventitious buds are not easy to die and grow rapidly in strong seedling culture. Therefore, obtaining the adventitious buds induced by the hypocotyl of Zikui tea and conducting growth culture first is conducive to the survival and growth of adventitious buds in strong seedling culture.

### Effect of dark culture on hypocotyl Browning

**Figure 9:**
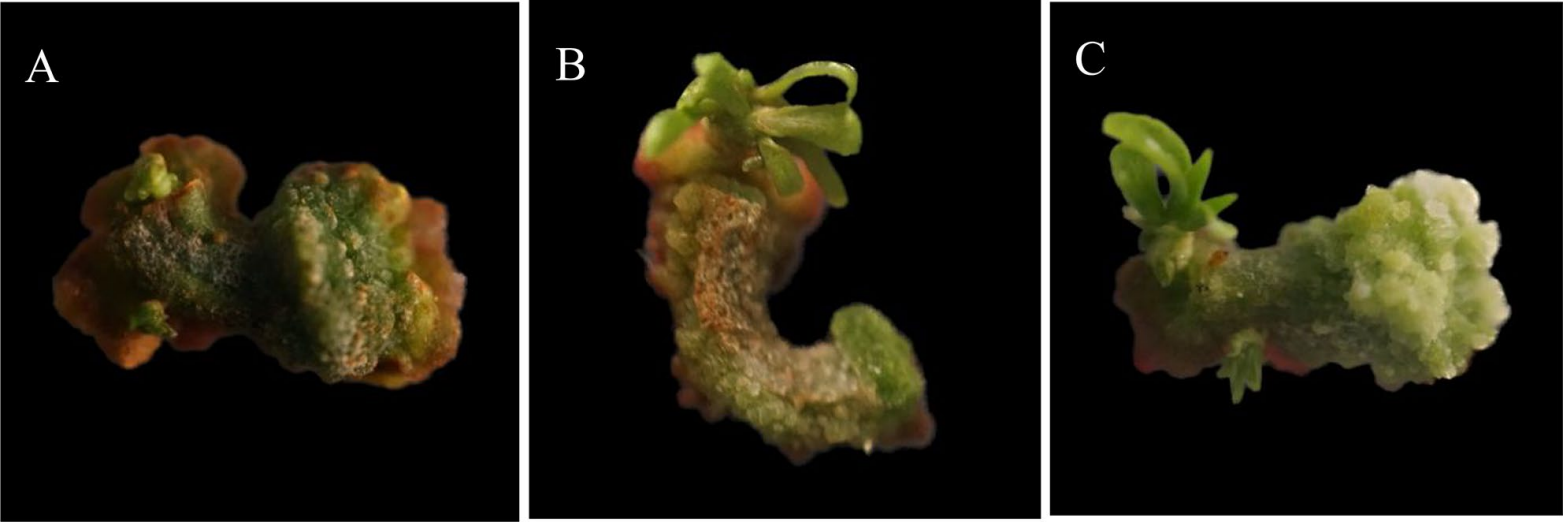
The condition of hypocotyl Browning in different dark culture days. (**A**) Browning of hypocotyl after 0 days of dark culture. (**B**) Browning of hypocotyl after 7 days of dark culture. (**C**) Browning of hypocotyl after 14 days of dark culture. The ruler in the figure is 1 cm.

Tea trees contain high levels of polyphenols, and the cut is oxidized to brown quinones. It poisoned explants in the medium and caused explants to brown and die. In this study, the effect of optimum dark culture days on the control of hypocotyl Browning is determined. Hypocotyl is cultured in the most suitable medium WPM + 2.00 mg/L + 6-BA + 0.10 mg/L NAA, and the Browning is observed (Table 5). The germination rate under normal light condition is higher after dark culture for 7 days and 14 days, and the Browning rate is 48.98%. It is difficult to differentiate buds (Fig. 9A). The Browning rate of dark culture for 7 days is 25.44%. The differentiated buds are weaker (Fig. 9B). The Browning rate of 14 days in dark culture was 9.93% (Fig. 9C). The difference is very significant, and the browning rate is much lower than that under normal light. It can be concluded that dark culture time is effective in inhibiting hypocotyl browning, and 14 d is the best dark culture time.

**Table 5:** Effect of dark culture days on hypocotyl callus formation and differentiation.

Dark cultivation time (d)	Number of vaccinations	Browning rate (%)	Browning number
0	90	48.98 ± 0.77a	47
7	90	35.44 ± 2.23b	34
14	90	19.93 ± 1.66a	19

### Effect of plant growth regulator on strong seedling

**Figure 10:**
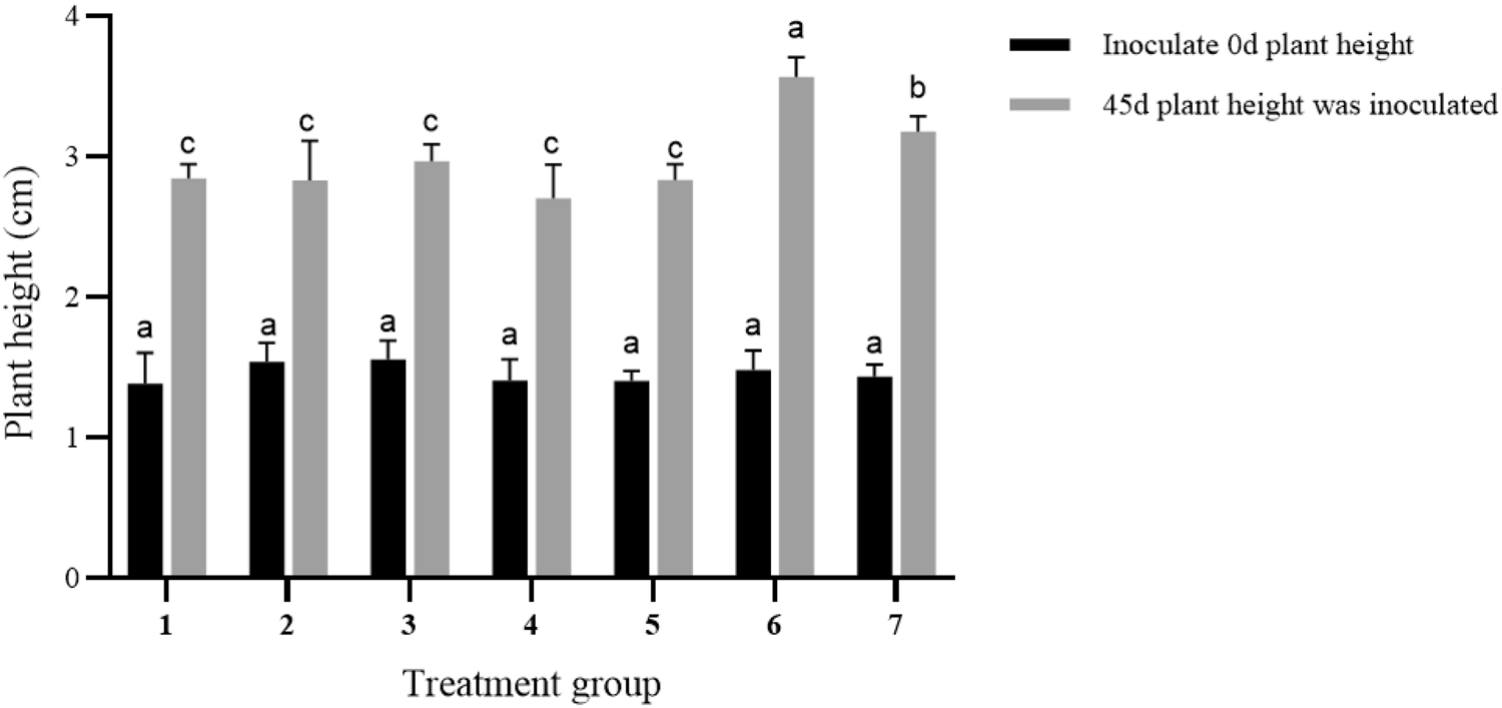
Effects of different plant growth regulators on strong seedling. Different letters on the bars indicate significant differences with each other (P < 0.05), while same letters indicate the lack of significance, as determined by one-way analysis of variance (ANOVA) with Duncan’s post-test.

**Figure 11:**
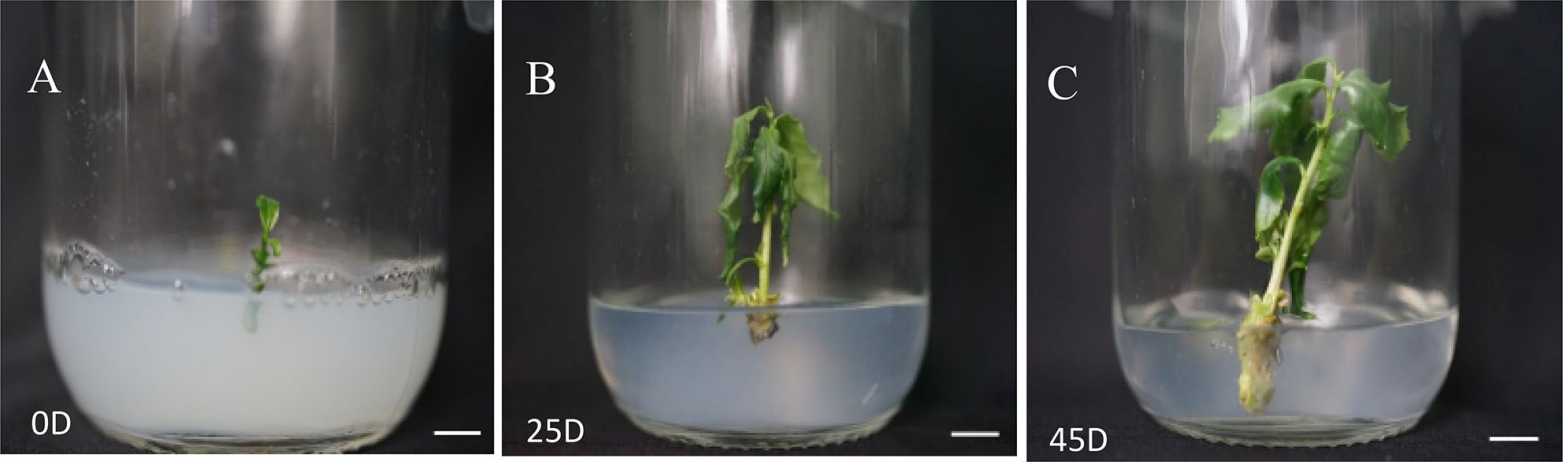
Growth of seedlings in strong seedling medium (**A**) seedling growth at 0 day in MS + 2.00 mg/L + 6-BA + 0.60 mg/L IBA seedling medium. (**B**) Seedling growth in MS + 2.00 mg/L 6-BA + 0.60 mg/L IBA strong seedling medium for 25 days. (**C**) Growth of axillary buds in MS + 2.00 mg/L 6-BA + 0.60 mg/L IBA seedling culture for 45 days. The ruler in the figure is 1 cm.

Strong seedling culture can enhance the plant robustness, thereby establishing a solid foundation for the subsequent rooting stages. Previous research demonstrated that varying ratios of plant growth regulators have a noteworthy impact on the strength of plant seedlings (Table 6). When 2.00 mg/L 6-BA + 0.60 mg/L IBA are added to the MS medium, the plant height difference reached 2.12 cm after 45 days, which is the highest among all treatments (Fig. 10). Therefore, this combination is considered as the optimal treatment (Fig. 11).

**Table 6:** Effects of different ratios of plant growth regulators on seedling strength.

6-BA(mg/L)	IBA (mg/L)	Inoculated number	Plant height difference (cm)	45 days plant growth status
–	–	120	1.51 ± 0.17c	The stem is thin and tall, with poor growth
1.20	–	120	1.28 ± 0.22c	Stem thin and short, poor growth
2.00	–	120	1.40 ± 0.10c	The stem is thin and short, the base has tufted bud differentiation, growing well
1.20	0.60	120	1.31 ± 0.03c	Stem thick and short, poor growth
1.20	0.90	120	1.45 ± 0.07c	The stem is thick and tall and grows moderately
2.00	0.60	120	2.12 ± 0.09a	The stem is thick and tall and grows well
2.00	0.90	120	1.81 ± 0.18b	The stem is thick and tall, the base has tufted bud differentiation, the growth is good

The difference in plant height is 1.51 cm when no hormone is used. However, when 2.00 mg/L of 6-BA is applied, the difference in plant height between the 6th and 7th treatment groups increased to 1.81 cm. Therefore, it can be concluded that 2.00 mg/L of 6-BA is the optimal concentration.

### Effect of plant growth regulators on plant rooting

**Figure 12:**
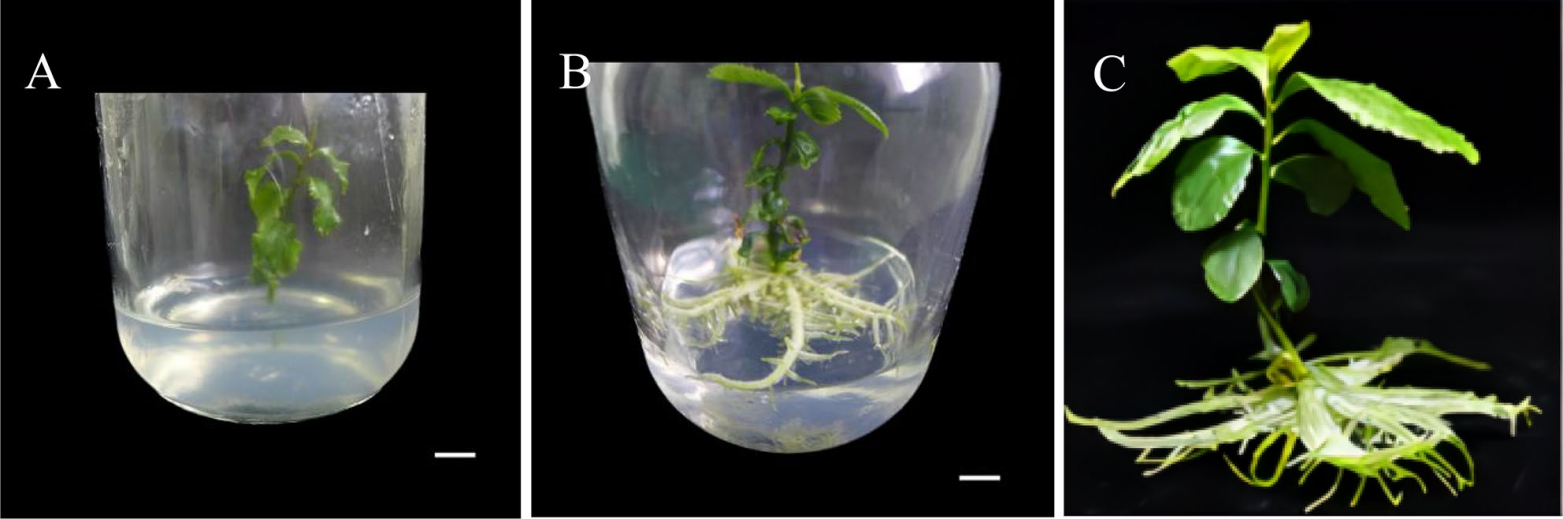
Rooting of plants in rooting medium for different days. (**A**) Growth of plants in 1/2 MS + 1.60 mg/L IBA rooting medium for 0 day; (**B**) Growth of plants in 1/2 MS + 1.60 mg/L IBA rooting culture for 45 days; (**C**) Growth of plants in 1/2 MS + 1.60 mg/L IBA rooting medium for 60 days. The ruler in the figure is 1 cm.

NAA and IBA are plant growth regulators that could be alone or in combination. They play a certain role in promoting plant cell division and promoting plant development. The results showed that the ratio of different plant growth regulators have a significant effect on plant rooting in tissue culture seedlings (Table 7). Under the condition of 1/2 MS + 1.60 mg/LIBA, the rooting rate after 60 days is 68.57%. These values are the highest among all the treatment groups (Fig. 12). Therefore, the medium suitable for rooting of tissue culture seedlings of Zikui tea tree wis 1/2 MS + 1.60 mg/LIBA.

**Table 7:** Effects of different ratios of plant growth regulators on plant rooting.

Processing condition	Inoculated number	Rooting rate (%)	Mean number of roots
1/2 MS	35	8.89 ± 1.65ef	2.00 ± 0.00f
1/2 MS + 0.50 mg/L NAA	35	26.67 ± 1.65d	3.67 ± 0.58e
1/2 MS + 0.50 mg/L IBA	35	30.48 ± 1.65c	6.33 ± 0.58c
1/2 MS + 1.60 mg/L NAA	35	56.19 ± 1.65b	7.33 ± 0.58b
1/2 MS + 1.60 mg/L IBA	35	68.57 ± 2.86a	8.67 ± 0.58a
1/2 MS + 0.25 mg/L IBA + 0.25 mg/L IBA	35	10.48 ± 1.65e	5.33 ± 0.58d
1/2 MS + 0.80 mg/L NAA + 0.80 mg/L IBA	35	6.67 ± 1.65f	4.33 ± 0.58e

### Effect of seedling refining time and transplanting medium on survival rate of plants

**Figure 13:**
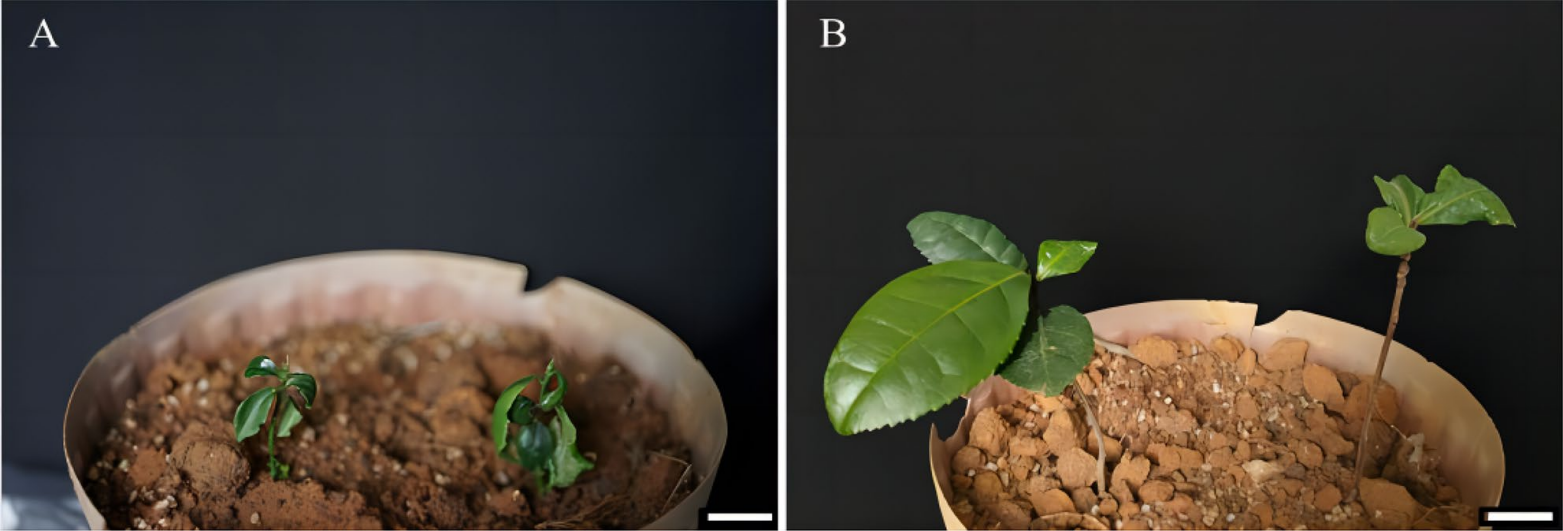
Living plant of Zikui. (**A**) The growth of plants in V yellow loam: V vermiculite = 2:1 in the transplanting medium for 0 day; (**B**) Growth of plants in V yellow loam: V vermiculite = 2:1 transplanting medium for 60 days. The ruler in the figure is 1 cm.

The results of the six sets of data (Table 8) indicated that the survival rate progressively increased as the seedling cultivation time increased. When the seedling cultivation time reached 6 days, the survival rate exceeded 59.42%, suggesting that this duration is considered suitable for seedling cultivation. The survival rate of V yellow loam with V vermiculite = 2:1 ratio is higher than that of V nutrient soil with V vermiculite = 2:1 ratio. When the ratio of V yellow loam: V vermiculite = 2:1 and seedling cultivation time was 6 days, the survival rate wis the highest (65.22%) among the 6 treatment groups. Moreover, under these conditions, the plants grew vigorously after transplantation, the leaves extended, and the roots continued to extend downward (Fig. 13). Therefore, the seedling cultivation time of Zikui was 6 days, and the transplanting medium is selected V yellow loam: V vermiculite.

**Table 8:** Effect of different time and medium on plant transplanting.

Seedling refining time (days)	Nutrient soil (2:1)	Survival rate (%)
4	Yellow loam: vermiculite	27.54 ± 2.51d
4	Nutrient soil: vermiculite	20.29 ± 2.51e
6	Yellow loam: vermiculite	65.22 ± 4.35e
6	Nutrient soil: vermiculite	59.42 ± 2.51b
8	Yellow loam: vermiculite	42.03 ± 2.51c
8	Nutrient soil: vermiculite	40.58 ± 2.51c

## Discussion

In the process of plant tissue culture, whether the aseptic system can be successfully established is the key to determine the whole experiment^19^. The woody plant tea tree is exposed to the external environment all the year round, and it is easy to epiphyte a variety of microorganisms, so it is necessary to establish a sterile system for the explants. The choice of disinfectant and the control of sterilization time are the key to reduce material contamination^20^. Studies have shown that hypochlorous acid can be oxidized to inactivate cells^21^. This has achieved good results in the disinfection of *Camellia sinensis* seeds and rosewood stem segments^22,23^. Therefore, 75% alcohol and 20% sodium hypochlorite are used for cross sterilization in this experiment. In this study, the optimal disinfection time of seed embryos is 75% alcohol for 3 min and 20% sodium hypochlorite for 10 min. Liao^24^ concluded that the explants of the same plant differ in their location, and the degree of disinfection is also different. Xie^25^ argued that the most suitable disinfection time is 12 min for stem segment of Zhonghuang No.1 tea tree and 20 min for seed embryo of Zhonghuang No.1 tea tree. Tian^26^ results showed that the most suitable disinfection time for the stem segment of Niaowang tea is 10 min disinfection with 10% sodium hypochlorite, and the most suitable disinfection time for Niaowang tea seed embryo is 15 min. This is different from the Zikui tea plant, the stem segment disinfection time is 20% sodium hypochlorite disinfection 13 min^27^, the seed embryo disinfection time is 20% sodium hypochlorite disinfection 10 min. It would be because Zikui tea tree is a new strain of purple leaf tea tree, and its seeds carry a large number of endophytic bacteria, resulting in a longer disinfection time, which is different from other varieties of tea trees.

In the process of callus induction and differentiation, proper dark culture is conducive to cell dedifferentiation and callus formation, while light inhibits callus formation. Based on our hypothesis, we found that dark culture could significantly reduce the Browning and callus induction of Zikui. Too long dark culture time will promote the growth of callus, too short time cannot produce adventitious buds^28^. When Zhang^29^ used rose leaves for callus induction, he found that after dark culture for 10 days and 12 days, he found that 12 days is the best culture time, and the induction rate of advective buds is 23.58%. Nie^30^ using tea seed hypocotyl as explants, the callus induction rate reached 44.43% after dark culture in callus induction medium for 4 weeks, and the explants without dark culture could not induce callus. Many studies have shown that light can improve the activity of peroxidase and thus increase the degree of Browning. It has been found that dark culture not only promotes callus induction, but also significantly reduces the Browning rate of explants. Liu^31^ found in the induction of mature embryos of maize that dark culture could significantly reduce the occurrence of Browning. Li^32^ studied the effect of light conditions on Browning by using stem segments of Populus chinensis as explants. The results showed that dark culture at the early stage of culture could effectively reduce the Browning rate, and dark culture for 10 days was appropriate. Xu’s^33^ experimental results also showed that the increase of light intensity would lead to the intensification of explants’ Browning. Woody plants have a very high content of phenols. The appropriate time of dark culture can lay an important foundation for the establishment of efficient regeneration system. The material used in this study is rare purple tea tree, which is rich in a lot of anthocyanins, so the degree of Browning during tissue culture is greater than that of other kinds of tea tree. However, the induction rate of callus is significantly higher than that of other woody plants, which may be due to the fact that dark culture effectively inhibited the release of anthocyanins, and the induction rate and differentiation rate showed an increasing trend.

In this study, GA_3_ is utilized to alleviate seed dormancy and enhance seed germination. Huang^34^ disinfected Campanula seeds and inoculated them into GA_3_ medium, and found that with the increase of GA_3_ concentration, the seed germination rate increased, and the germination rate reached 82.40% in MS + 1.00 mg/L GA_3_ medium. Wu^35^ found that the low concentration of GA_3_ promoted the pollen germination of Fuding white tea, but when the concentration of GA_3_ reached 50.00 mg/L, the pollen germination was inhibited. In this study, after removing the cotyledon, the germination time of Zikui tea embryo was longer in MS blank medium. When GA3 is added to the medium, the germination time was shorter, and the germination index and cotyl length are increased. This is consistent with previous studies that gibberellin can promote the germination of tea seeds^36,37^. When GA_3_ concentration is 3.60 mg/L, the germination rate decreased slightly, which may be due to high or low plant hormones affecting the germination and growth of tissue culture seedlings^38^. Higher concentration of GA_3_ also promoted bud differentiation, thus inhibiting the growth length of the cotyl. The results of this study are consistent with the results of Yang^39^ on the adverse effects of high GA_3_ concentration on seed germination of Toona sinensis.

Common basic media for tea tree culture include MS, 1/2 MS, WPM, B5, White, N6, ER, MT, etc. MS medium has the characteristics of complete trace elements and high inorganic salt content. It is used in the process of axillary bud germination^40^. Axillary bud proliferation, callus induction and differentiation of explants^41^. In existing reports, MS medium is the most commonly used in tea plant tissue culture^42^. Therefore, in this experiment, MS medium was used as the base medium in the process of germination, proliferation and seedling strengthening.

WPM medium was designed by Lloyd and McCown in 1981 for the culture of mountain laurel stem tips, modified from MS medium, compared with MS medium, the content of ammonium nitrate is reduced to 1/4 of MS, in which the nitrogen salt is also supplied in the form of calcium nitrate. Fan^43^ found that the formula was WPM + 2.00 mg/L 6-BA + 1.50 mg/L NAA in the young embryo culture of camotea, and the seedling formation rate reached 90.00%, while the embryogenic callus induction of immature cotyledon was WPM + 1.00 mg/L 6-BA + 0.50 mg/L NAA. Therefore, in this experiment, WPM medium was also used as the base medium in the callus formation and differentiation stage of the cotyl. After adding appropriate plant growth agents, the callus induction rate could reach more than 88.89%.

In this experiment, at the rooting stage, 1/2 MS as the base medium had the best effect, with lower sucrose concentration and salt concentration and higher NH_4_NO_3_ concentration than other medium, which inconsistent with the view that tea tree is an ammoniophilic plant with high NH_4+_ absorption rate. NAA and IBA are synthetic plant growth regulators that can be administered alone or in combination to induce root formation and development. The experimental study showed that the rooting rate was very low when NAA and IBA were mixed, which may be related to the inhibiting effect of NAA and IBA mixed on the rooting of tea tree. This is consistent with Zuo^44^ finding that “Shancha No. 1” had a very low rooting rate in the medium of 0.50 mg/L IBA and 0.50 mg/L NAA IBA alone is also found to be more beneficial to rooting than NAA. Studies have shown that soaking tea plants with auxin before rooting can effectively shorten the rooting initiation time^45^. Li^42^ selected robust bacteria-free vaccine of tea tree, soaked it in 500.00 mg/L IBA for 2 min, and then inoculated it into the rooting medium. Fu^46^ also used 500 g/L IBA to soak Huangshan Kucha inoculum for 10 min before inoculation to rooting medium. In this experiment, before inoculation into the rooting medium, 60.00 mg/L IBA sterile solution is soaked for 8 min, and good rooting effect is obtained under the condition of 1/2 ms + 1.6 mg/L IBA, which is consistent with the results of previous studies.

Anthocyanins are important secondary metabolites, widely found in land plants. Zikui tea plant is rich in anthocyanins, about 50 times that of other tea plants. It is one of the model plants for the study of anthocyanins. Some studies have shown that the biosynthesis of anthocyanins in plants is largely dependent on light. However, the regulatory mechanism of light-induced anthocyanin accumulation remains unclear^47^. Chen^48^ found that anthocyanins can determine the coloring of nightshade, and selected light-insensitive nightshade materials. Wang found that the color of turnip is induced by ultraviolet light, UV-A monochromatic light, and blue + UV-B composite light, possibly because photoinduction is a response to anthocyanin synthesis in plants. Jiang found that the anthocyanin content of uninduced solanum japonica was 81% of that of normal Solanum Japonica. In this study, the buds of Zikui did not exhibit the typical purple characteristics in plant tissue culture, possibly because they were not induced by light. You^49^ found that the content of anthocyanins in purple tea bud leaves is closely related to the depth of color of the bud leaves, which may be because of the role of chlorophyll, making the color darker. In this study, it was found that the color of the leaves of Zikui during tissue culture was dark green instead of pure purple, which may be due to the interaction between chlorophyll and anthocyanins, which is consistent with the conclusion you^49^ reached. The content of anthocyanins in the plant is much higher than that of other plants, and the leaf color of the tea plants is more-dark green in tissue culture than that of other tea plants after the interaction of chlorophyll.

In this study, the tissue culture method is employed to investigate the Zikui tea tree embryo as explants. The focus is on determining the optimal sterilization conditions and induction differentiation medium for the explants. The study also examined the optimal conditions for transplanting and refining adventitious shoots. Specifically, the research focused on understanding the regeneration system of the Zikui tea plant, which can offer valuable insights for future genetic transformation studies on Zikui tea plant genes.

## Conclusion

This study successfully developed an efficient regeneration system using the hypocotyls of Zikui tea as explants. Throughout the cultivation process, optimal treatment conditions and appropriate medium formulations are achieved. An efficient regeneration system with stable hypocotyl as explants is established, which laid an important regeneration method for genetic transformation technology of Zikui.

## Data Availability

All data are availables in the manuscript.
